# Association of vitamin D levels and oral lichen planus. Systematic review and meta-analysis

**DOI:** 10.4317/medoral.26603

**Published:** 2024-06-22

**Authors:** Sonia Egido-Moreno, Joan Valls-Roca-Umbert, F Javier Parra-Moreno, Enric Jané-Salas, Andrés Blanco-Carrión, José López-López

**Affiliations:** 1 Department of Odontoestomatology, Faculty of Medicine and Health Sciences, School of Dentistry, University Campus of Bellvitge, University of Barcelona, Barcelona, Spain; 2 Master of Oral Medicine, Surgery and Implantology, Medicine and Health Sciences Faculty, UB, Barcelona, Spain; 3 Oral Health and Masticatory System Group, IDIBELL (Bellvitge Biomedical Research Institute), University of Barcelona, Barcelona, Spain; 4 Oral Medicine, Oral Surgery and Implantology Unit (MedOralRes), School of Medicine and Dentistry, University of Santiago de Compostela, Santiago de Compostela, Spain; 5 Faculty Director and Head of Service of the Medical-Surgical Area of Dentistry Hospital, University of Barcelona, Barcelona, Spain

## Abstract

**Background:**

Oral lichen planus (OLP) is an inmuno-mediated mucocutaneous chronical inflammatory disease. Multiple predisposing factors are considered, such as autoimmune response, microorganisms, medications, dental materials, psychological stress, genetic predisposition or nutritional deficiencies. The deficiency of vitamin D has been related to various autoimmune diseases like OLP.

**Material and Methods:**

The electronic search was conducted in the MEDLINE (Pubmed), Scopus, Cochrane Library and Web of Science databases. To assess any potential risk of bias, the authors critically appraised each study by the Newcastle-Ottawa Scale for cohort and case-control studies. Pooled analyses were performed using a random-effects model. Heterogeneity of the studies was assessed by the I2 statistics. Forest Plots were performed to graphically represent the difference between vitamin D concentrations in the OLP compared to healthy group, with a 95% confidence interval.

**Results:**

After applying our inclusion and exclusion criteria, 7 articles were included in our review. The median concentration vitamin D in ng/ml found in serum for patients with OLP was of 26,6311,75ng/ml and for healthy patients was of 31,438,7ng/ml. Regarding the quantitative analysis, 7 studies were included. The difference in the concentration of vitamin D in healthy patients and patients with OLP statistically significant (Weighted Mean Difference (WMD): -6.20, 95% CI: -11.24 to -1.15, *p*=0.02 and I2 heterogeneity: 94%, *p*<0.00001).

**Conclusions:**

The patients with OLP have statistically lower vitamin D levels than healthy patients.

** Key words:**Vitamin D, oral lichen planus, hypovitaminosis D, vitamin D deficiency.

## Introduction

Oral lichen planus (OLP) is an inmuno-mediated mucocutaneous chronical inflammatory disease. Its prevalence is of 0.5% to 2% and most commonly found in women older than 40 years of age (1,2). The OLP may affect skin, genital and oral mucosa. Implication of the oral mucosa is frequent, between 15-35% of the cases it is the only place affected by the disease (3).

Clinically, the OLP’s most common way of expression is by greyish-whitish striae (Whickam’s striae), and it can also be found as erosive, atrophic, ulcerated or even blistering forms (1,4).

OLP manifestations usually continue for years, alternating between periods of latency and exacerbation (3,5). The initial diagnosis of OLP must be based in the recognition of its clinical manifestations (6,7), the diagnostic confirmation must be achieved through a histopathological study, and to differentiate the disease from other entities with similar clinical appearance (7).

Recent studies have reported that the malignant transformation rate of OLP ranges from 0.44% to 2.28% (3). Warnakulasuriya *et al*. (8) included OLP as a potentially malignant disorder, and despite it being of lower risk (7), many investigators recommend the indefinite monitoring of patients with OLP in order to detect early stages of malignant evolution (5). The time span of the disease and the age of the patient is related to the chances of malignant evolution; this could be related to the continuous chronical inflammation and be the base for this malignant evolution (7).

Even though the etiopathogenesis is still unknown, it is widely accepted that the development of the lesions is related to a T cell response against epithelial cells (9). Multiple predisposing factors are considered, such as autoimmune response, microorganisms, medications, dental materials, psychological stress, genetic predisposition or nutritional deficiencies (3).

Vitamin D is said to play an important role in the immune system. Vitamin D receptors (VDRs) are found on immune cells which include B cells, T cells, and antigen-presenting cells (10). It is a lipid-soluble vitamin that is mainly ingested in the form of vitamin D2 and D3 via dietary intake and exposure to sunlight. Both forms of vitamin D are metabolized and activated to 25-hydroxyvitamin D (25(OH)D) in the liver, and subsequently converted to the active form of vitamin D, 1, 25-dihydroxyvitamin D3 (1,25(OH)2D3), in the kidney. As the main circulating metabolite in the blood, 25(OH) D is the most representative indicator of vitamin D storage in the human body (11).

The deficiency of vitamin D has been related to various autoimmune diseases such as systemic lupus erythematosus, oral lichen planus, insulin dependent diabetes mellitus, inflammatory bowel disease, multiple sclerosis, and rheumatoid arthritis (12).

Even more, Vitamin D has been demonstrated to have anticancer properties, including the prevention of cancer cells’ angiogenesis, metastasis, and invasiveness. Numerous *in vitro* and *in vivo* studies on various cancer types have demonstrated the anticancer properties of calcitriol. Vitamin D insufficiency has been linked to an increased risk of head and neck cancer, especially oral squamous cell carcinoma and oral potentially malignant disorders (13).

The aim of this systematic review is to answer the question: Do patients with OLP have lower concentrations of vitamin D than healthy patients? Therefore, the following PECO (Population, Exposure, Comparison, Outcome) question was designed: In a group of patients whose vitamin D (P) concentrations are analyzed, are there differences in the concentration of vitamin D (O) between patients with OLP (E) and healthy patients?

## Material and Methods

This systematic review was conducted following the Preferred Reporting Items for Systematic Reviews and Meta-analyses (PRISMA) (14). A detailed protocol was prepared before starting the review and registered on Prospero (ID:498063)

- Information sources

The electronic search was conducted in the MEDLINE (Pubmed), Scopus, Cochrane Library and Web of Science databases. Also, the references of the studies selected for eligibility were revised for potential inclusion. The last search was performed on December 31th, 2023.

- Eligibility criteria

Inclusion criteria were articles in which the level of vitamin D in OLP subjects and healthy subjects was compared. Criteria for inclusion were randomized clinical trials (RCT), non-randomized clinical trial, observational studies (cohort and case-control) and cross-sectional studies. Studies where the diagnosis of OLP has been made through clinical histopathological criteria.

No restriction was made for the year of publication and English or Spanish language.

The exclusion criteria were articles that investigated the association of VDR and OLP gene polymorphisms or the pathways of OLP lesions due to VDR deficiency; studies focused on cutaneous lichen planus. Articles that do not provide with the diagnostic method of OLP or the diagnosis has only been achieved through clinical exploration. Case series, case reports or reviews were excluded.

- Study selection

Two authors (SEM, JVR) independently reviewed the abstracts of all references localized in the databases. Studies were selected if they appear to meet the inclusion criteria according to their abstracts. The same authors independently revised the full texts of the articles identified in the initial selection and screened the references of these studies to identify others for potential inclusion. Disagreements between reviewers were resolved by consensus by two other authors (EJS and JLL). A Cohen kappa for each database was calculated to determine the interrater reliability.

- Data extraction

One author (SEM) extracted the data from the selected studies for qualitative synthesis: authors name, publication year, country, study design, population, sample size, study groups, age and gender of the included studies, analytical marker, medium, detection technique, follow-up period, exposure/intervention, primary outcome, outcome measures, main results, and the test of significance. Two authors (EJS and JLL) checked the extracted data.

- Outcomes

Primary outcome was incidence/risk of vitamin D deficiency in OLP of vitamin D (clinical appearance or subjective symptoms).

- Risk of bias in individual studies

To assess any potential risk of bias, the authors critically appraised each study by the Newcastle-Ottawa Scale for cohort and case-control studies (15).

- Synthesis of results

Pooled analyses were performed using a random-effects model. Heterogeneity of the studies was assessed by the I2 statistics. Heterogeneity among studies was considered statistically significant for a *p-value* < 0.05 and was interpreted as recommended by the Cochrane Handbook: 0%- 40% was considered unimportant, 30%-60% as moderate heterogeneity, 50%-90% as substantial heterogeneity, and 75%-100% as considerable heterogeneity. The Review Manager 5.4 program was used as a tool to analyze the data, previously recorded in an Excel Table. Forest Plots were performed to graphically represent the difference between vitamin D concentrations in the OLP compared to healthy group, with a 95% confidence interval (CI).

## Results

- Study selection

**Figure 1:**
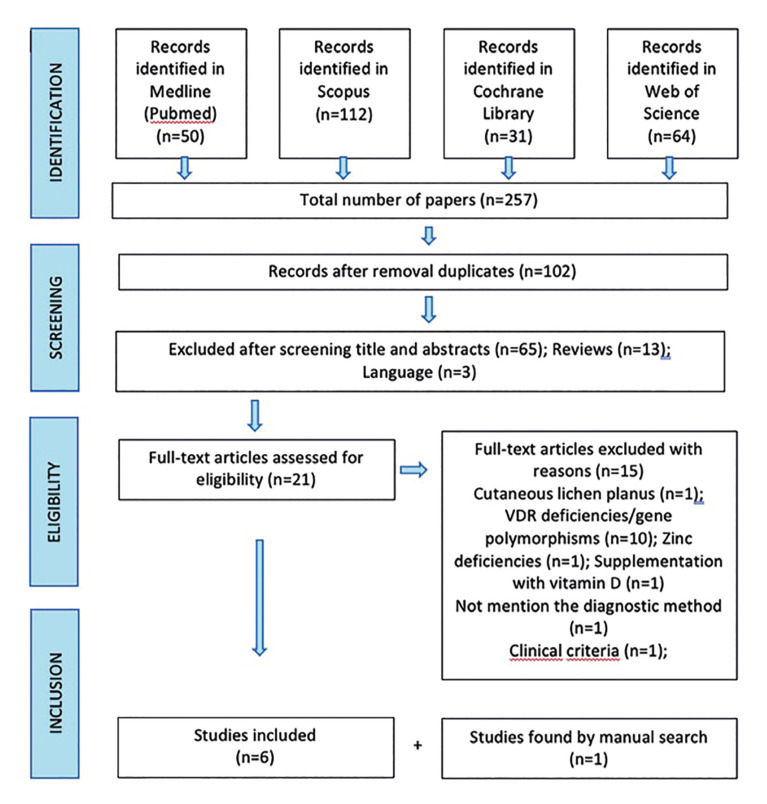
Flow chart.

A total of 257 articles were obtained using our search strategy. After duplicated were removed, 102 articles were screened for eligibility. After abstracts assessment 62 records were excluded, 13 for being a review and 3 for being in language other than English or Spanish. 24 articles were selected for full text assessment. The Cohen kappa was 0.89 for Medline/Pubmed, 0.73 for Scopus, 1 for Cochrane Library and 1 for Web of Science. Of these, a total of 18 articles were excluded as they failed to meet the inclusion criteria; 1 reported outcomes of cutaneous lichen planus, 10 articles focus on VDR deficiencies or gene polymorphisms, one (16) because it did not mention the diagnostic method of the OLP and another one (17) because they achieved the OLP diagnosis through clinical criteria. One study (12) reported outcomes about treatment with vitamin D supplementation, the consensus by two reviewers finally decided to exclude this article. One article (18) was added by manual search. Finally, seven (18-24) articles were included in the review for the qualitative synthesis. Fig. 1.

- Study characteristics

All the studies included were in English. The characteristics of the studies are summarized in Table 1. The included articles were cross sectional case-control studies. Four articles were conducted in Iran (18,19,21,24), one in India (20), one in Egypt (22), and one in Croatia (23). The total population included 559 patients, of whom 299 were diagnosed with OLP versus 260 of healthy patients. The diagnosis of OLP was achieved through clinical and histopathological examination (18-20,22-24) and in one study it was specified that WHO criteria for the diagnosis of OLP were followed (21).

With regards to the gender, five articles gave information about the sex in the OLP group as in the non OLP group (18,19,22,23,24), one article only gave gender information in the OLP group (20) and 1 study did not give any information about the gender of the participants (21). In the OLP group 72,77% (*n*=171) were women and 27,23% (*n*=64) were men. In the control group 64,89% (*n*=131) were women and 35,11% (*n*=54) were men.

With regards to the age of the participants, in 1 study (21) no information was given, in another article (20) the median age of the participants was given without any group distinction, and on the remaining 5 articles (18,19,22-24) both median ages for the OLP group and the control group were given, which were of 51,844,47 and 49,014,51 years respectively.

Regarding to the technique used to determine the concentrations of vitamin D, 3 articles (21,22,23) used Enzyme-linked immunosorbent assay (ELISA), 1 article (23) used Electrochemiluminescence immunoassay (ECLIA), one study (18) High-Performance Liquid Chromatography (HPCL), one article (19) used electrochemiluminescence technique and finally one article (20) used the electrochemiluminescence binding assay.

The determination of the concentrations of vitamin D were detected in serum in six studies (18-20,22-24) and in 1 study (21) it was determined in serum, non-stimulated saliva and stimulated saliva. The articles (19-22,24) that provide with the concentration of vitamin D in serum, the results are measured in ng/ml. In the study by Družijanicì *et al*. (23) the result is measured in nmol/L, which by the conversion factor 2,496 we translated to ng/ml, in order to better compare the results. In the study by Rezazadeh *et al*. (18) the concentration in serum for healthy patients was of 50.136.47ppm, while in OLP patients was of 46.994.27. The median concentration vitamin D in ng/ml in serum for patients with OLP was 26,6311,75ng/ml and for healthy patients was of 31,438,7ng/ml. The study from Gholizadeh *et al*. (21) provided data for the vitamin D concentration in saliva. For the non-stimulated saliva, a result of 0,450.05ng/ml in OLP patients and 0,620,07ng/ml in healthy patients. For the stimulated saliva, a concentration of 0,430,05 and 0,630,07 in patients with OLP and healthy, respectively.

- Risk of bias

The risk of bias across the studies is presented in Table 2. All studies have a score below 7, therefore all have a high risk of bias.

- Meta-analysis

**Figure 2:**
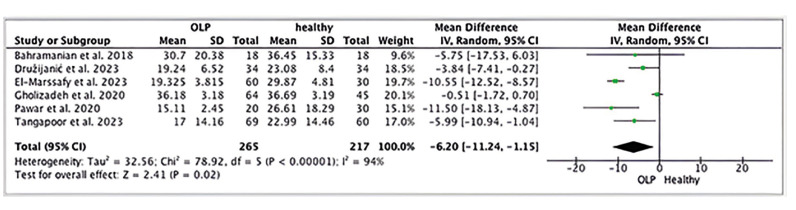
Forest plot.

Regarding the quantitative analysis, six studies (19-24) were included. The study of Družijanicì *et al*. (23) provided us with the results in nmol/L which we converted to ng/ml through the conversion factor of 2,469 in order to homogenize the results and compare them. In the Forest Plot a graphical representation is provided for the difference in the concentration of vitamin D in ng/ml in serum of both healthy patients and patients with OLP. The study from Rezazadeh *et al*. (18) was not included in the metaanalysis since it did not provide with the results in universal units for vitamin D and the comparison between the results is not possible. The difference in the concentration of vitamin D in healthy patients and patients with OLP in statistically significant (Weighted Mean Difference (WMD): -6.20, 95% CI: -11.24 to -1.15, *p*=0.02 and I2 heterogeneity: 94%, *p*<0.00001). Fig. 2.

## Discussion

This systematic review and metaanalysis are aimed to evaluate the relation between deficiency of vitamin D and the OLP.

The global prevalence of OLP has been assessed to be about 1.01% (25). This frequency is greater in women and even more frequent in the perimenopausal (10.91%) than in premenopausal women (0.5-2.0%). It is considered that declined levels of estrogen and progesterone can directly or indirectly (that can be determining factors for the onset of depression as well) cause OLP (26). This data coincides with our review, given the greater percentage (72,77%) of OLP was present in women.

Micronutrients, including trace elements, vitamins, and antioxidants, play an important role in regenerative processes against oxidative stress products in the tissues. Many investigators have noticed the association between micronutrient deficiency and inflammatory disorders. levels of iron and its associated markers such as hemoglobin and ferritin, increased level of TIBC, reduced levels of zinc, calcium, vitamin D, vitamin B12, folic acid and antioxidants such as vitamins C and E, and increased levels of oxidants and homocysteine have been reported in OLP patients (27).

The role of vitamin D in the regulation of the immune responses has been widely accepted. It acts in the OLP by means of i. Antiinflamatory and immunomodulation action, vitamin D produces the expression of antimicrobial peptides such as the defensins β2 y β4 cathelicidin antimicrobial peptide. These antimicrobial properties stimulate the body’s defense mechanism against microbial infections. Vitamin D modulates the adaptive immune response as much as the innate. Calcitriol exhibit a downregulatory effect on the cell-mediated (Th1) immune responses by suppressing the release of type 1 proinflammatory cytokines (such as IL-6, IL-8, IL-12, IL-17, IL-21, IFN-γ, TNF-α, and IL-9). However, it upregulates the humoral (Th2) response by facilitating the production of type 2 anti-inflammatory cytokines (such as IL-4, IL-5, and IL-10) (3). ii. Differentiation and proliferation of keratinocytes. Although with a higher prevalence of vitamin D deficiency, up-regulation of miR-346 and TNF-α and down-regulation of vitamin D receptor were reported to be responsible for the induction of apoptosis in oral mucosal keratinocytes in OLP patients (3,27,28). iii. Adrenal cortisol regulation. A higher amount of anxiety, depression and psychic ailments have been found in patients with OLP. The chronical stress, attributed as the major predisposing factor for acute outbakes of OLP, triggers a higher production of suprarenal cortisol and provokes a decrease in the expression of vitamin D receptors (3).

In our study, the concentrations of vitamin D have been significantly lower in patients with OLP than in healthy patients. This coincides with other reviews such as Motahari *et al*. (29) in which they find the odds of vitamin D levels in patients with OLP being lower than 30 ng/ml were 2.65 times higher than those in the control group

A possible correlation between the severity of OLP and the magnitude of the vitamin D deficit has been mentioned. The study by El Marssafy *et al*. (22) in which a lower concentration of vitamin D was found in patients with erosive/symptomatic OLP in comparison with patients with non-erosive/symptomatic OLP.

The supplementation of vitamin D has also been studied. Nazeer *et al*. (12) provided a supplement of vitamin D (60,000 IU weekly) to patients with OLP and vitamin D deficit, as an adjuvant treatment to topical steroids and observed a significant improvement of the symptomatology and clinical signs. Nevertheless, in the review from Saeed *et al*. (3) they conclude that despite the vitamin D supplements might improve the symptoms of OLP, there is still no definitive protocol, due to the lack of knowledge about the pathogenesis of OLP. Other reviews such as Dave *et al*. (10) studied the association of LP to other systemic diseases and found that a greater number of cases were receiving a treatment with vitamin D supplements, and those patients medicated with vitamin D supplements were 2.5 times more likely to be diagnosed with than those without supplement treatment. The effects of the vitamin D supplementation have also been studied. In the review by Mataurana-Ramírez *et al*. (30) a relationship between hypovitaminosis D and the malignant transformation of potentially malignant lesions in the oral cavity due to alterations in the immune response. As well as being associated to a lesser survival rate for patients with higher recurrence rate of tumors in patients that undergo surgical treatment and an increase in adverse reactions to chemotherapy.

The vitamin D deficit is considered a concentration lower than 30ng/ml. In our review the levels of vitamin D were 26,6311,75ng/ml for patients with OLP and of 31,438,7ng/ml for healthy patients. The levels found are below the levels considered normal (of 30-100 ng/ml), in patients with OLP and in the lower limit for healthy patients. Currently the vitamin D deficiency is a condition prevalent in both develops and undeveloped countries. This may be due to a variety of reasons, including increased use of sunscreen, increased indoor activity, and more skin coverage due to different cultural beliefs and religious practices or protection against skin cancer (29).

All the studies in this review used serum to study vitamin D (18-23), even though recently saliva has been proposed as the medium to obtain biomarkers, since it is less invasive and easier to obtain, such as it has been demonstrated in the study by Gholizadeh *et al*. (21).

This systematic review and metaanalysis has some limitations, such as the studies that have been included not having a low risk of bias, which could lead to errors in the conclusions and high heterogeneity.

## Conclusions

The patients with OLP have statistically lower vitamin D levels than healthy patients. The median levels of the participants, both healthy and with OLP, are lower than what is considered normal. It would be recommended a routinely checkup of the vitamin D levels in these patients.

## Figures and Tables

**Table 1:** Characteristics of the studies included.

Author/ Year/ Country	Type of study	Diagnosis OLP	Parameter Technique Serum/ saliva	Control			OLP			Conclusion
				n (F/M)	Age ± ds [range]	Mean ± ds vitamin D	n (F/M)	Age ± ds [range]	Mean ± ds vitamin D	
Bahramian et al (19), 2018, Irán	Case-control study	Clinical examination and histopathologic analysis	25-OH-D Electrochem iluminescence technique Serum	18 (14/4)	49.94 [21-76]	36.45±15.33 ng/ml	18 (11/7)	44.16 [30-71]	30.7±20.38 ng/ml	No statistically significant difference *p=*0.346
Družijanić *et al.* (23), 2023, Croatia	Case-control study	Clinical examination and histopathologic analysis WHO criteria	25-OH-D ECLIA Serum	34 (26/8)	47 [18-79]	57.7±21 nmol/L	34 (29/5)	56.5 [21-84]	48.1±16.3 nmol/L	Statistically significant lower serum vitamin concentrations in patients with OLP compared to control group. *p=*0.001
El-Marssafy *et al.* (22), 2021, Egypt	Case-control study	Clinical examination and histopathologic analysis	25-OH-D ELISA Serum	30 (24/6)	47.1±7.71	29.87±4.81 ng/ml	60 I: (24/6) II: (25/5)	I: 47.47±6.39 II: 48.70±7.20	I: 23.14±5.28 ng/ml II: 15.51±5.77 ng/ml	Statistically significant difference between OLP patients (group I and II) and the control group (group III) *p=*0.000
Gholizadeh *et al.* (21), 2020, Iran	Case-control study	Clinical examination and histopathologic analysis WHO criteria	25-OH-D ELISA Serum and SS/US	45 -	-	Serum: 36.69 ± 3.79 ng/ml US: 0.62± 0.07 ng/ml SS: 0.63± 0.07 ng/ml	64 -	-	Serum: 36.18 ± 3.18 ng/ml US: 0.45±0.05 ng/ml SS: 0.43±0.05 ng/ml	Serum: 0.267 US: 0.001 SS: 0.026
Tangapoor *et al.* (24), 2023, Iran	Cross sectional study	Clinical examination and histopathologic analysis	Vitamin D_3_ ELISA Serum	60 (42/28)	51.01 ± 13.74	22.99 ± 14.46 ng/ml	69 (48/21)	55.33 ± 11.60	17.00 ± 14.16 ng/mL	The mean serum levels of vitamin D in patients with OLP were significantly lower than in healthy subjects *p=*0.000
Pawar *et al.* (20), 2022, India	Case-control study	Clinical examination and histopathologic analysis	25-OH-D Electrochem iluminescence binding assay Serum	30 -	45.5 [18-80] (total study population)	26.10± 18.29 ng/ml	20 (11/9)	45.5 [18-80] (total study population)	15.11± 2.45 ng/ml	Significant lower levels of vitamin D3 in OLP patients. p<0.05
Rezazadeh *et al.* (18), 2021, Iran	Case-Control study	Clinical examination and histopathologic analysis	Vitamin D3 HPLC Serum	43 (25/18)	48.744 ± 12.70	46.99 ± 4.27 ppm	34 (23/11)	48.031 ± 11.57	50.13 ± 6.47 ppm	Lower level of Vitamin D in OLP patients, but not statistically significant *p=*0.802

OLP: Oral Lichen Planus; ECLIA: Electrochemiluminescence immunoassay; ELISA: Enzyme-linked immunosorbent assay; I: Assymptomatic OLP (reticular, papular and plaque-like lesions); II: Symptomatic OLP (atrophic or bullous-erosive lesions); US: unstimulated saliva; SS: Stimulated saliva; HPCL: High-Performance Liquid Chromatography * (>30ng/ml defined as normal level, 20-29ng/ml categorized as insufficient, 10-19ng/ml as moderately deficient.

**Table 2:** Quality assessment for case-control and cohort studies based on New Castle-Ottawa Scale.

	Selection	Comparability	Exposure	Score (0-9)
Bahramanian *et al.* (19)	★★★	★	★★	6 High risk of bias
Družijanić *et al.* (23)	★★★	★	★★	6 High risk of bias
El-Marssafy *et al.* (22)	★★	★	★★	5 High risk of bias
Gholizadeh *et al.* (21)	★★★	★	★★	6 High risk of bias
Tangapoor *et al.* (24)	★★★	★	★★	7 High risk of bias
Pawar *et al.* (20)	★★	★	★	4 High risk of bias
Rezazadeh *et al.* (18)	★★	★	★	4 High risk of bias
